# Retracted: Combined Case of Blood-Injury-Injection Phobia and Social Phobia: Behavior Therapy Management and Effectiveness through Tilt Test

**DOI:** 10.1155/2016/9802939

**Published:** 2016-09-18

**Authors:** Case Reports in Psychiatry

Case Reports in Psychiatry has retracted the article titled “Combined Case of Blood-Injury-Injection Phobia and Social Phobia: Behavior Therapy Management and Effectiveness through Tilt Test” [1]. The article was found to contain a substantial amount of material from published articles, some without citation, including the following:

Heimberg RG et al.: Psychometric properties of the Liebowitz Social Anxiety Scale. Psychological Medicine, 1999, 29, 199–212 (not cited);

Ayala ES, Meuret AE, Ritz T: Treatments for blood-injury-injection phobia: a critical review of current evidence. J Psychiatr Res. 2009 Oct; 43(15):1235-42. doi: 10.1016/j.jpsychires.2009.04.008;

Lars-Göran Öst, Ulf Sterner: A specific behavioral method for treatment of blood phobia. Behaviour Research and Therapy Volume 25, Issue 1, 1987, Pages 25–29 doi: 10.1016/0005-7967(87)90111-2;

Lars-Göran Öst, Anita Jerremalm, Jan Johansson: Individual response patterns and the effects of different behavioral methods in the treatment of social phobia. Behaviour Research and Therapy. Volume 19, Issue 1, 1981, Pages 1–16 doi: 10.1016/0005-7967(81)90107-8;

Ryan C. Shorey and Gregory L. Stuart: Manualized Cognitive-Behavioral Treatment of Social Anxiety Disorder: A Case Study. Clin Case Stud. 2012 Feb 1; 11(1): 35–47. doi: 10.1177/1534650112438462 (not cited).
